# On the combinatorics of crystal structures. II. Number of Wyckoff sequences of a given subdivision complexity

**DOI:** 10.1107/S2053273323002437

**Published:** 2023-05-11

**Authors:** Wolfgang Hornfeck, Kamil Červený

**Affiliations:** a FZU – Institute of Physics of the Czech Academy of Sciences, Na Slovance 1999/2, 182 00 Prague 8, Czechia; Universidad del País Vasco, Spain

**Keywords:** Wyckoff sequences, combinatorics, Shannon entropy, structural complexity

## Abstract

Wyckoff sequences are a way of encoding combinatorial information about crystal structures of a given symmetry. In particular, they offer an easy access to the calculation of a crystal structure’s combinatorial, coordinational and configurational complexity, taking into account the individual multiplicities (combinatorial degrees of freedom) and arities (coordinational degrees of freedom) associated with each Wyckoff position. However, distinct Wyckoff sequences can yield the same total numbers of combinatorial and coordinational degrees of freedom. In this case, they share the same value for their Shannon entropy based subdivision complexity. The enumeration of Wyckoff sequences with this property is a combinatorial problem solved in this work, first in the general case of fixed subdivision complexity but non-specified Wyckoff sequence length, and second for the restricted case of Wyckoff sequences of both fixed subdivision complexity and fixed Wyckoff sequence length. The combinatorial results are accompanied by calculations of the combinatorial, coordinational, configurational and subdivision complexities, performed on Wyckoff sequences representing actual crystal structures.

## Abstract 2

The number of Wyckoff sequences of a given subdivision complexity is calculated by means of a generating polynomial approach and a dynamic programming approach. The result depends on the choice of space-group symmetry (which is obligatory) and Wyckoff sequence length (which is optional). It also takes into account specified values for the total number of combinatorial and coordinational degrees of freedom, thereby representing crystal structures of invariant subdivision complexity.

## 1. Introduction

Any standardized crystal structure can be conveniently related to a descriptor uniquely encoding its combinatorial properties, namely its *Wyckoff sequence*: a string composed of the space group type number (sometimes the Hermann–Mauguin symbol is used instead) and followed by all the Wyckoff letters for each partially or fully occupied Wyckoff position in the crystal structure; the letters are put in reverse alphabetic order and augmented by their superscripted frequency of occurrence, in case a certain non-fixed Wyckoff position is occupied multiple times.

Standardization is necessary because many crystal structures do have equivalent descriptions in terms of their unit cell and atomic coordinates, depending either on matters of possible unit-cell choices or the symmetry properties of space groups [keywords: symmetry of symmetry, Cheshire groups, Euclidean normalizers; see Müller (2013 ▸), ch. 8]. A comprehensive scheme for crystal structure standardization has been theoretically developed by Parthé and coworkers (TYPIX) and implemented into the software *STRUCTURE TIDY* (Parthé & Gelato, 1984 ▸, 1985 ▸; Gelato & Parthé, 1987 ▸; Parthé *et al.*, 1993*a* ▸,*b* ▸). The uniqueness of the Wyckoff sequence thus depends on and follows from the uniqueness of the standardization.

The Wyckoff positions of a space group encompass all possible distinct sets of symmetry-equivalent sites within a unit cell. Or, to put it in rigorous mathematical terms: a Wyckoff position of a space group 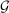 consists of all points *X* for which the site-symmetry groups 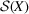 are conjugate subgroups of 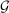 (see Hahn, 2005 ▸, ch. 8.3.2, p. 733; Aroyo, 2016 ▸, ch. 1.4.4.2, p. 62).

It is important to distinguish the notions of space group and space-group type. A space group encompasses all of the symmetry of an actual crystal structure, including its translation symmetry, specified by its lattice parameters. A space-group type is an abstract notion comprising all those, otherwise distinct, space groups which share the same representation by matrix-column pairs of symmetry operations (Nespolo *et al.*, 2018 ▸). This is of special importance in the study of group–subgroup relations, in which a group and one of its proper subgroups can be of the same space-group type, but not of the same space group. Another consequence is that the number of space groups is infinite, whereas the number of space-group types is finite: 230 distinct ones in three dimensions, including 11 enantiomorphous types. In particular, the Hermann–Mauguin symbol represents the space-group type, while concepts related to the Wyckoff positions, such as the Wyckoff sequences studied in this work, are related to the space group, in the sense that they are inseparably connected with the coordinate description of crystal structures. However, the number of distinct Wyckoff positions is considered to be finite, with a total of 1731 positions for all space groups, thus allowing for the combinatorial analysis to follow.

The Wyckoff positions are labelled by up to 27 possible Wyckoff letters (*a* to *z* and α), being devoid of any further meaning, but depending on the choice of space group. Since the Wyckoff sequence composed of these letters does not include any specific information about either the unit-cell parameters or the atomic coordinates, it is a more abstract notion as well, and useful as such for purposes of crystal structure classification and systematics (see, *e.g.*, Allmann & Hinek, 2007 ▸). Note that this abstraction means that actual crystal structures can share the same Wyckoff sequence, while being distinct with respect to the specific values of the free parameter(s) of their non-fixed Wyckoff position(s). Such crystal structures are called isopointal (Lima-de-Faria *et al.*, 1990 ▸).

Yet, Wyckoff sequences can be studied without referring to specific geometric crystal structures. Thus, any Wyckoff sequence really describes an infinite family of crystal structures, sharing a common parametrization in terms of their geometric degrees of freedom, according to the observed partial or full occupancy of their general position(s) and, if present, special position(s) of their corresponding space-group type. Indeed, the Wyckoff sequence has been used in the aforementioned sense, as a coordinate-free representation of crystal structures in modern machine learning approaches to materials discovery (Goodall *et al.*, 2022 ▸).

One particular advantage of this abstract, combinatorial point of view is due to the fact that the number of Wyckoff sequences of given length *k* is finite (Hornfeck, 2022*a* ▸), making an exhaustive study possible for small values of *k*. In fact, most of the actual crystal structures found so far in nature have Wyckoff sequences of length below *k* = 50. For the space-group type *Pmmm* (No. 47) with ν = 19 non-fixed sites and φ = 8 fixed ones, constituting the case with the highest number of possible sequences, this would correspond to about 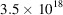 distinct sequences to consider in total, up to the length of *k* = 50 inclusive (see Appendix *A* [App appa] for the computation and the exact result).

Another advantage, and one focus of this work, is due to the fact that the Wyckoff sequence translates into the information about a crystal structure’s combinatorial (*M*) and coordinational (*A*) collective degrees of freedom, associated with a weighted sum of each Wyckoff position’s individual multiplicity ( 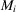 ) and arity ( 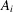 ), respectively, the latter being the number of independent coordinate parameters required to be specified in a standardized description of an actual crystal structure.

Both the combinatorial and coordinational degrees of freedom can be used to assess a crystal structure’s combinatorial and coordinational complexity by means of an approach pioneered by Krivovichev (2012 ▸, 2014 ▸) and extended by Hornfeck (2020 ▸, 2022*b* ▸) based on the utilization of the Shannon entropy as a complexity measure.

Taking a general point of view, the collective degrees of freedom represent a certain system (a macrostate), while the individual degrees of freedom each represent a certain subsystem (a microstate). This is true on different levels of hierarchy. For instance, on the crystal structure level, the collective configurational degrees of freedom, 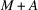 , result as the sum of the individual combinatorial (*M*) and coordinational (*A*) degrees of freedom. In a similar way, on the Wyckoff position level, the collective combinatorial and coordinational degrees of freedom, *M* or *A*, result as the sum of the individual combinatorial and coordinational degrees of freedom, 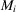 or 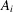 , respectively.

In combinatorial terms this splitting of a system into subsystems corresponds to an integer partition. However, in their crystallographic application, the number of partitions is not simply given by the number-theoretic partition function, yet restricted by crystallographic symmetry and the coupling of combinatorial and coordinational degrees of freedoms as found within each Wyckoff position. In particular, the possible values of the Wyckoff multiplicities considering all space-group types are restricted to the set 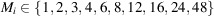 (assuming primitive unit cells, *i.e.* modulo centring translations, which, in the following, will be the preferred choice of description), while the possible values of the Wyckoff arities are restricted to the set 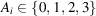 . Moreover, for a given choice of space-group type, the values of either set that can occur are restricted further (although not their frequencies of occurrence, except for fixed positions, which can occur only once), in accordance with the existing Wyckoff positions. A final restriction is imposed by each Wyckoff position introducing a coupling of values from both sets. The combinatorial problem under consideration thus is one of counting the number of restricted, coupled partitions.

Thus, due to these particular restrictions, it is a non-trivial task to describe a macrostate defined by the collective degrees of freedom 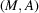 by the composition from or the subdivision into its associated microstates 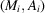 . Foremost, it is a natural question to ask, given one macrostate, what is the number of microstates corresponding to it?

The following section will give the answer to this question, with the main results being a generating polynomial approach (Section 2.4, theory; Section 2.7, algorithm) and a dynamic programming approach (Section 2.8, theory and algorithm).

## 2. Combinatorics of Wyckoff sequences

To state the problem succinctly: how many distinct Wyckoff sequences (of optionally fixed length *k*) exist, given a pair of combinatorial and coordinational degrees of freedom 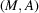 together with a choice of space group? (Note that a space group has to be fixed, since this determines the alphabet of Wyckoff letters which can appear in the Wyckoff sequence.)

### 2.1. A problem of crystals – exposition

The problem statement can be translated rather straightforwardly into some algebraic form. Note that two integer values exist for each individual Wyckoff position *i*, its multiplicity 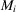 and its arity 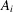 (coordinational degree of freedom). Both have to be considered in a coupled way, which will be done in the notation by the use of column vectors. Then, the total multiplicity and arity, *M* and *A*, respectively, corresponding to a certain set of Wyckoff sequences of crystal structures, are given as 

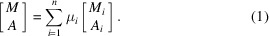

Here, the sum index *i* runs over all the *n* existing Wyckoff positions for a given space group, with the integer multipliers 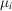 denoting the frequency of occurrence of a given site in the sum of individual multiplicities 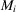 and arities 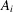 of the combined sites. In one general point of view, as mentioned in the *Introduction*, one can call the pair 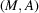 the system variables (describing the macrostate), while the 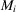 and arities 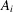 would be the subsystem variables (describing the microstates).

Information about the Wyckoff positions of the 230 three-dimensional space-group types is compiled in Vol. A of the *International Tables for Crystallography* (Aroyo, 2016 ▸), which is the authoritative source. Alternatively, it can also be retrieved online from the Bilbao Crystallographic Server (https://www.cryst.ehu.es/) using the routine *WYCKPOS* (Aroyo *et al.*, 2006*a* ▸,*b* ▸, 2011 ▸).

The values of the Wyckoff multiplicities 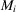 are explicitly stated as the numeral part of the Wyckoff symbol assigned to each Wyckoff position (the non-numeral part is given by the Wyckoff letter). Note, however, that these values are given for the centred unit cells, in which case *M* should also be specified with respect to the unit-cell content of the centred unit cell, in order to maintain the correct correspondence. Alternatively, the values of the Wyckoff multiplicities can be reduced by the division of an integer factor depending on the centring type (2 for *C*, *A* and *I* centring; 3 for *R* centring; 4 for *F* centring), if the primitive unit cell, and the number of atoms it contains, is taken as a reference. Indeed, choosing the primitive unit cell as a reference is strongly recommended in the context of crystallographic complexity calculations (*cf*. Section 3), in order to make the results of these calculations comparable for crystal structures differing in their centring type, since any existing centring translation just repeats parts of a crystal structure inside a unit cell, thereby contributing no additional information to its description, and, accordingly, its complexity.

The values of the Wyckoff arities 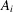 , although not explicitly stated in the aforementioned sources, can be deduced from them, namely by means of visual inspection with respect to the number of positional variables *x*, *y* and *z* occurring in the listing of the general coordinates as provided for each Wyckoff position.

In general, this information can be obtained for any crystallographic space group, including higher-dimensional ones, namely by inspecting the number of symmetry-equivalent positions modulo translations, to obtain the multiplicities, as well as by elucidating the dependency of their general coordinates with respect to symmetry, to obtain the arities. In fact, all this information can be obtained in a purely algorithmic manner [see Brown *et al.* (1978 ▸) for the case of four dimensions].

**Figure 1:**
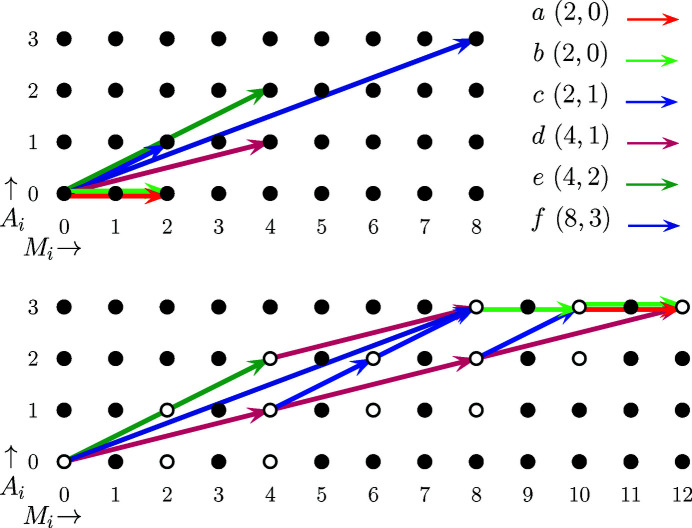
Geometric interpretation for the problem of finding the number of ways of combining individual Wyckoff positions of given multiplicities and arities, 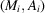 , adding up to a given total multiplicity and arity 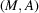 . In the illustration, the target vector 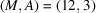 , here written in row form, is denoting a point in the two-dimensional integer lattice 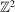 (square lattice) in the upper-right corner. On the top, the individual vectors 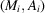 corresponding to each Wyckoff position are shown: *a* (2,0) red, *b* (2,0) green, *c* (2,1) blue, *d* (4,1) dark red, *e* (4,2) dark green, *f* (8,3) dark blue. On the bottom, their combinations adding up to 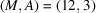 are shown, with vectors composed in reverse lexicographic order. Other possible combinations, in which only the order of the vectors are changed, are not shown. However, all lattice points which can be reached by any possible combinations of vectors are highlighted as open circles instead of filled ones. To see the full graph one has to invert the depicted half of it in the point (6, 1.5). In this interpretation the problem becomes a special case of a lattice path enumeration problem with the set of steps governed by the Wyckoff multiplicities and arities for a given choice of space-group symmetry.

It should be noted that the problem at hand can be given different mathematical interpretations and representations. Equation (1) already suggests a geometrical interpretation as two-dimensional vectors, which happen to live on the two-dimensional integer lattice 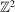 (square lattice). The problem then appears as a special case of a lattice path problem (Fig. 1 ▸), in which the vectors associated with the Wyckoff positions of a space group define the set of steps.

Alternatively, the two-dimensional plane can be identified with the complex plane of Wessel, Argand and Gauß suggesting a change of notation, in which *i* is the imaginary unit ( 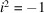 ): 

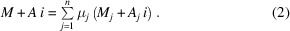




While these interpretations and representations are mathematically equivalent, and thus do not seem to make any difference *per se*, the knowledge of these alternatives can be of importance when it comes to searching for subfields of mathematics discussing already existing solutions to a given problem, or general methods to find them, and also in the case of implementation into computer code.

### 2.2. Conditions on the multipliers

For any given site the multipliers 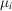 are restricted to discrete intervals 

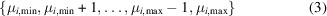

of potential integer values, which are denoted in shorthand as 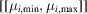 in the following. Naturally, by definition, the frequencies of occurrence are bounded from below, likewise for all sites, by all minimal multipliers 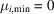 , meaning that a site is absent in this case. The variable upper bounds, the maximal multipliers, are determined according to the case distinction 

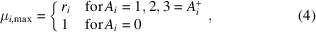

differentiating between non-fixed and fixed sites. Collecting the terms for the non-fixed and fixed sites separately, the summation of equation (1) can be split into separate parts,

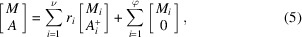

in which ν and φ now denote the total number of non-fixed and fixed sites, respectively (compare Hornfeck, 2022*a* ▸).

In any case, the repetition 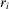 is given as 

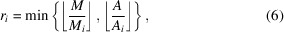

with 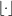 denoting the floor function, and the smaller ratio restricting the number of possible occurrences of the corresponding site from above. Stated in a different way, each multiplier for any non-fixed site has to fulfil both of the conditions 

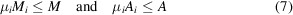

simultaneously, with 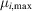 being defined as the largest integer doing so.

These conditions originate from the fact that the Wyckoff multiplicities and arities are non-negative integers; thus any surpassing of either one of the limits *M* or *A* cannot be balanced by the addition of another Wyckoff position, hence cannot be a part of the solution, and thus signifies an end to the Wyckoff sequence construction process. This guarantees the existence of maximal values 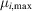 and fixes the size of the search space of which the solution space is a subspace.

While the determination of the size of the solution space, the number of Wyckoff sequences existing for a given choice of 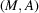 , is our main task, the determination of the size of the search space appears as a first step towards a result, in that it gives a numerical upper bound for the size of the solution space, a combinatorial overview about the Wyckoff sequences to expect, with respect to their length, as well as an estimate regarding the computational tractability of their actual construction based on an exhaustive exploration of the search space.

### 2.3. Size of the search space

For non-fixed sites the multiplier intervals are given as 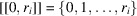 , with variable 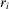 , while for the fixed sites the multiplier intervals are given as 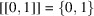 , invariably. The Cartesian product over all interval sets of multipliers of either type determines the search space:

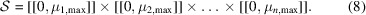

This corresponds to the set of all possible Wyckoff letter sequences, being Wyckoff sequences without the space-group type symbol/number prefix explicitly stated, as constructed from the multiset 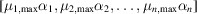 , in which the 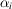 denote a general Wyckoff letter out of *n* possible letters for a given space group. Note that the choice of space group, while defining the alphabet of Wyckoff letters and limiting the number of terms to consider in the Cartesian product, does not determine the size of the search space by itself. This size, the cardinality 

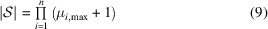

of the search space, in which individual solutions have to be found, if they exist, is determined by the values of the maximal multipliers. Thus, the size of the search space is determined only if both the space group and the total numbers of degrees of freedom *M* and *A* are fixed. Then, the search space size gives an absolute upper bound on the potential number of solutions, yet usually, and in anticipation of our following results, the actual number of solutions will be much lower or even zero. This difference in the number of solutions is due to the construction of the search space by means of the Cartesian product, namely because the restrictions imposed by the 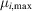 values for individual Wyckoff positions do not take into account their cumulative, conditional interactions.

As is often the case in combinatorial problems, the same cardinality 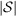 can be obtained by an alternative counting method, namely as the result of the summation of all coefficients in the expansion of the univariate polynomial defined by 

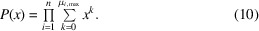

This is the generating polynomial for a multiset with finite multiplicities [compare its description and, in particular, equation (4) in Hornfeck (2022*a* ▸)]. Upon its expansion the coefficients 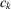 for each term 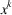 of this polynomial count the number of sequences of length *k*, thereby representing a more differentiated view of the search space’s contents. Eventually, 

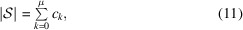

in which 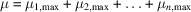 is the multiset’s cardinality.

The size of the search space gains some importance due to the fact that the combinatorial approach we will describe in the remainder of this work is non-constructive, as is commonly the case for such combinatorial questions, since it gives only the number of potential Wyckoff sequences matching with a given parameter pair 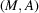 , but does not reveal the Wyckoff sequences themselves; these can be discovered by an exhaustive check of all admissible multiplier combinations existing within the combined multiplier intervals.

We envision that the search space size can be reduced to some degree by applying effective intermediate checks for multiplier combinations already violating the upper limit as imposed by the choice of the parameters 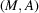 , overshooting either one parameter at a time or both simultaneously, possibly in combination with the use of a clever data structure such as pruned trees. However, search space sizes below 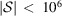 are easily tractable on a standard desktop personal computer, which should encompass most tasks related to the comparison of the potential combinatorial solutions with Wyckoff sequences representing actual crystal structures.

### 2.4. A generating polynomial approach to find solutions

Our combinatorial problem stated above is solved in two steps: first, by reducing it, conceptually, to an analogous classical problem of combinatorics, the coin change problem, as can be found in many textbooks on the topic (for instance, Marcus, 1998 ▸, p. 89), and second, by adapting the classical problem to the crystallographic one.

The classical coin change problem is stated in Appendix *B* [App appb], and can be seen as an illustration of the use of generating polynomials, defined in some abstract variable. Notably, the variable is an indeterminate symbol only, entailing no specific meaning other than to allow algebraic operations performed on it; hence it merely acts as a placeholder and bookkeeping device, yet is the decisive one, in order to systematically find a solution.

This use of generating polynomials has already been described in the first entry of this series (Hornfeck, 2022*a* ▸) to which the reader, interested in more detailed information, is referred. An introduction to the wider field of generating functions is given by Graham *et al.* (1994 ▸), and a more detailed exposition of the main ideas involved is given by Wilf (2006 ▸).

Now, adapting the classical coin change problem to our crystallographic one is carried out in three steps: (i) matching the number of distinct types of coins with the number of distinct Wyckoff positions; (ii) identifying the values of distinct types of coins with the *pair* of values of the Wyckoff position’s multiplicity 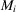 and arity 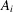 ; and (iii) treating the pair of values ( 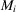 , 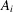 ) in a coupled way, by introducing two abstract variables 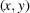 in the generating polynomials, instead of one.

Thus, in some way, the crystallographic problem is a coin change problem with a twist, based on imaginary coins with denominations on both their front and back sides and a pair of target values to reach upon summation. Similar combinatorial problems arise for the case of real cards, coupling values denoted by digits or letters with symbolic ones such as diamonds, hearts, spades and clubs.

In particular, the third adaptation step is crucial for the correct enumeration. As a consequence of it, generating polynomials of the kind 

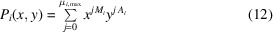

are assigned to each Wyckoff position, with their product over all *n* Wyckoff positions, 

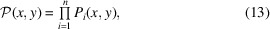

yielding the solution, namely by the value of the coefficient of 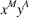 in the expanded form of the polynomial 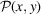 .

As an aside, one can note that the product over all *n* sites can be split, 

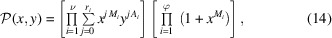

according to the contributions of ν non-fixed and φ fixed sites [compare equation (5)]. This splitting reduces the problem for the fixed sites to a univariate one.

It should be noted that this approach can be further generalized, in principle, by taking into account chemical degrees of freedom [as introduced by Hornfeck (2020 ▸)] in terms of atomic numbers of atoms occupying a given Wyckoff position as well. Then, one would have to introduce a third variable *z* into the respective polynomials with all other procedures considered to be analogously performed. However, there is a difference, in that the atomic numbers are not restricted in any way, that is to say there exists no natural coupling between them and the Wyckoff multiplicities and arities – they are a pure matter of choice. In contrast, the Wyckoff multiplicities and arities are coupled in their values for each Wyckoff position of a space group. Thus, regarding this relative arbitrariness of choice and the relative unimportance of this general case, we refrain from expanding in this direction for the moment. However, our opinion on this topic might change in the future, if there should be an interesting application for fixing the total chemical degrees of freedom, that is the total electron count within a reduced unit cell, to a set value, together with the other degrees of freedom, and asking for the number of crystal structures fulfilling this condition. In this case, any generalization required can be obtained in a straightforward manner by following the same extension procedure as described in the following.

### 2.5. A generalization including the Wyckoff sequence length

Another and considerably more useful generalization is made by taking into account the length *k* of the Wyckoff sequence as a further restriction, which can be seen as an extension and a refinement to a previous result in the combinatorics of Wyckoff sequences (Hornfeck, 2022*a* ▸). This can be achieved by adjusting equation (1) according to 

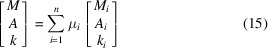

in addition to 

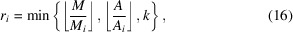

used for the determination of the 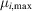 for each individual site *i*. Trivially, each site itself is of length 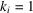 (thus, 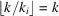 ), which gives the total length of the Wyckoff sequence as the sum of the multipliers 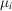 : 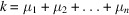 . Again, the final result is given as the value of the coefficient of 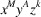 in the expanded polynomial 

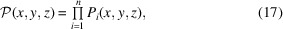

in which 

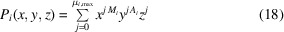

defines the individual terms.

As was the case before, the product over all *n* sites 

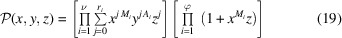

can be split according to the contributions of ν non-fixed and φ fixed sites.

Finally, it should be noted that there exist general methods for obtaining explicit formulas for the coefficients of generating functions, for instance for the powers of bivariate generating functions (Kruchinin *et al.*, 2021 ▸), as this is an active field of mathematical research.

### 2.6. A problem of crystals – exemplification

In the following, an illustrative example is discussed in full calculational detail.

The tetragonal space-group type 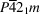 (No. 113) encompasses a total of six distinct Wyckoff positions, 

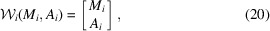

here written in a more convenient in-line notation, indexed according to their Wyckoff letters, and with their corresponding multiplicities and arities stated: 

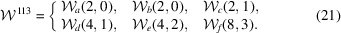

The chosen space group is the one with the smallest number of Wyckoff positions for which all possible arity values 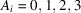 are present. The six Wyckoff positions comprise a total of three and four distinct values for the Wyckoff multiplicity and arity, respectively, thereby allowing us to illustrate the combinatorial calculation in some detail.

Now, with some arbitrary chosen 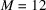 and 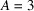 given for this particular example, this results in the following maximal multipliers:

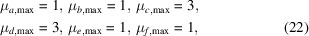

for each Wyckoff position, and consequently in the following generating polynomials:

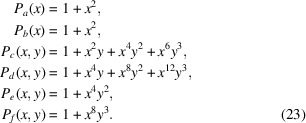

Note how the two fixed sites with Wyckoff letters *a* and *b* give rise to exactly the same polynomial factor in one variable *x* only (namely the one used for the Wyckoff multiplicities), and how the polynomials corresponding to the non-fixed sites are restricted in terms of the monomials of highest degree in either *x* or *y* by the given values of either *M* or *A* or both.

Note also that the number of variables in each polynomial corresponds to the splitting of all *n* sites into ν contributions from the non-fixed sites (bivariate case) and φ contributions from the fixed ones (univariate case) [compare equation (5)].

The expanded polynomial 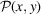 consisting of a total of 60 terms is given by 

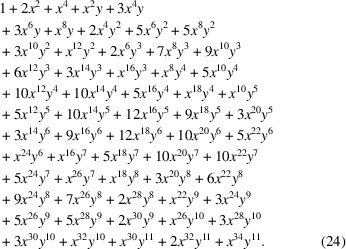

The decisive term 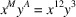 has 6 as its coefficient, which thus corresponds to the six existing solutions, looked for in the search space; they can be stated in terms of their Wyckoff letter sequence, with multiple occurrences of the same letter marked by superscripts: 

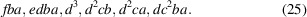

Note, in particular, the cases 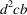 and 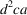 which differ only in the exact choice of the fixed site, being otherwise degenerate in their column vector summation [compare equation (1)].

In fact, the bivariate polynomial given in equation (24) contains the information about *all* possible solutions 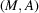 which can be constructed from the multiset of Wyckoff letters 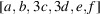 with restricted multiset multiplicities, as determined by the maximal multipliers. Since the multiplier intervals are 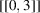 for both the Wyckoff positions *c* and *d*, as well as 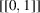 for all other sites, the search space has a size of 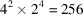 cases, whose distribution according to the length *k* of the sequence can be read off from the expansion (11 terms; not shown) of the generating polynomial 

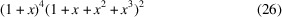

since 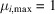 for four out of the six Wyckoff sites and 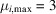 for the remaining two. The observed six solutions with the property 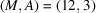 constitute only a tiny fraction of the search space, which is generally true, the size of the search space typically being overwhelmingly larger than the number of solutions.

In particular, the full set of solutions contains the singular occurrence of the empty sequence, 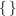 , corresponding to the trivial monomial 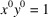 , as well as that of the maximal length sequence, *abcccdddef*, corresponding to the monomial 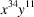 of highest degree, for which 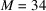 and 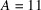 . Note how one can instantly check that there exists no solution for, say, the case 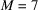 and 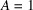 , because no monomial of the form 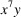 appears in 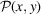 , or, to put it another way, the coefficient of this monomial in the expansion of 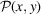 is equal to zero. Note also how one specific solution, namely solution 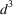 , corresponds to the occurrence of the monomial 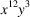 , the 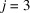 case of the basic monomial 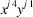 representing the 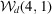 Wyckoff position, in the generating polynomial 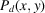 of equation (23).

Taking into account the length *k* as an additional parameter, one has to adjust the individual polynomial terms according to 

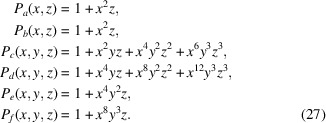

Expansion of their product yields a polynomial in 142 terms (not shown), with, for instance, the value of the coefficient for 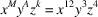 being equal to 3, corresponding to the Wyckoff letter sequences 

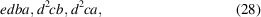

which form a subset of the six aforementioned solutions.

### 2.7. Summary of the generation function approach

To summarize the aforementioned results in an algorithmic form, using the generating polynomial approach one has to perform the following steps to obtain a solution (with the first two items in the enumeration defining the input to the algorithm and the last one its output):

(i) Fix a space group and thereby the set of Wyckoff positions and the potential alphabet of Wyckoff letters from which a Wyckoff sequence can be formed.

(ii) Fix the values for the total Wyckoff multiplicity *M* and the total Wyckoff arity *A* and (optionally) the Wyckoff sequence length *k*, all of which are expected to be integers.

(iii) Retrieve all the individual values for the Wyckoff multiplicities and arities 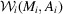 .

(iv) From the individual Wyckoff multiplicities and arities compute the individual maximal multipliers 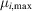 .

(v) From the individual maximal multipliers construct the individual generating polynomials, 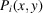 or 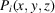 .

(vi) From the individual generating polynomials build their product, 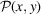 or 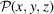 , and expand it.

(vii) From the expansion read off the coefficient for the monomial of the form 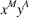 or 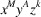 ; this is the solution.

### 2.8. A dynamic programming approach to find solutions

**Figure 2:**
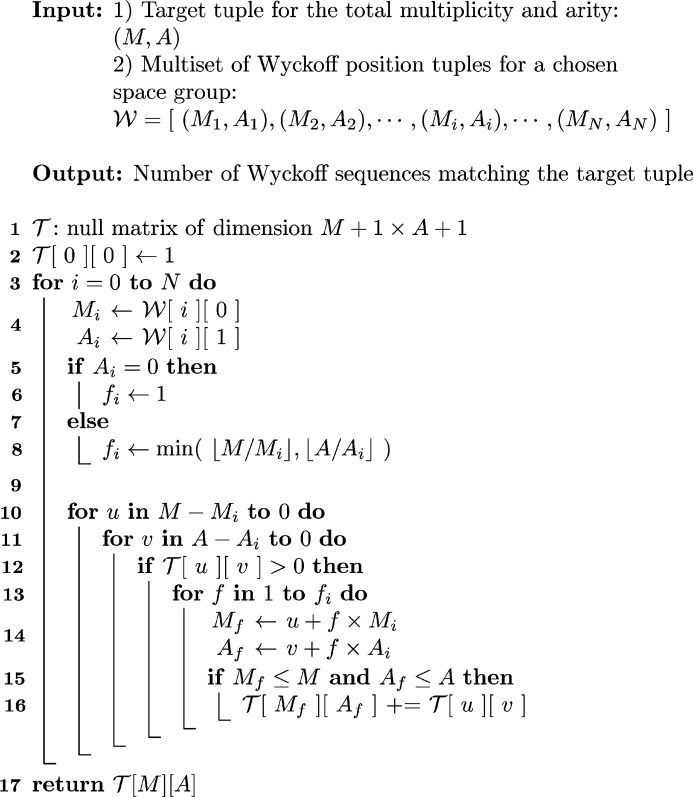
Dynamic programming algorithm (given in pseudocode) for the determination of the number of Wyckoff sequences of a given space group and subdivision complexity as determined by the total number of degrees of freedom for the Wyckoff multiplicity and arity (bivariate case).

**Figure 3:**
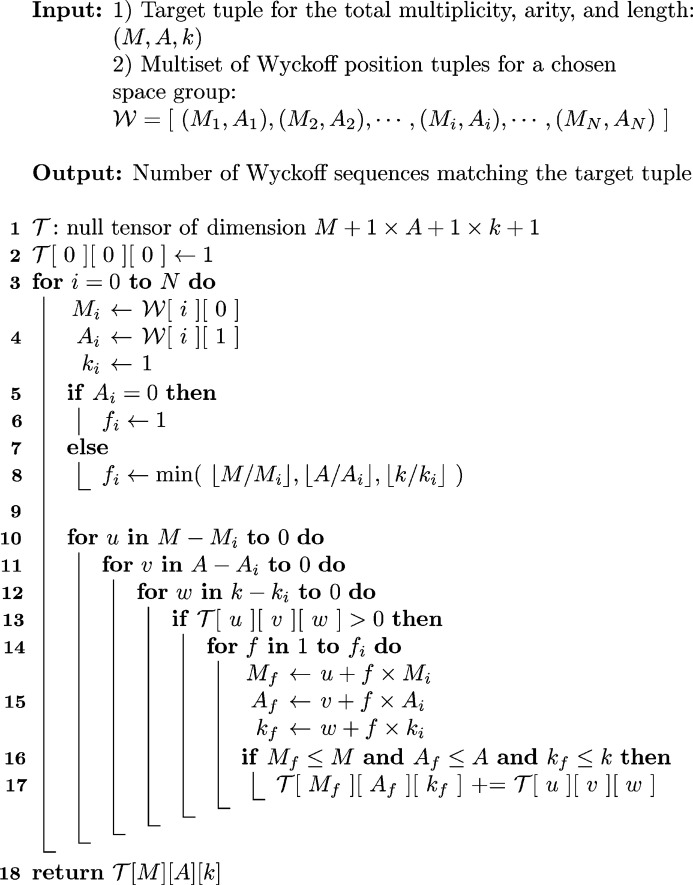
Dynamic programming algorithm (given in pseudocode) for the determination of the number of Wyckoff sequences of a given space group, subdivision complexity and length as determined by the total number of degrees of freedom for the Wyckoff multiplicity, Wyckoff arity and length (trivariate case). Note that 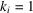 for all Wyckoff sites – this does not have to be specified for each Wyckoff position independently. However, possible simplifications due to this fact have not been included in the pseudocode in order to highlight its similarity with the bivariate case shown in Fig. 2 ▸.

Finally, after illustrating the aforementioned combinatorial method on an explicit example, we want to highlight the possibility of an alternative algorithmic way of calculation which turns out to be very efficient, by making optimal use of the recursive nature and overlapping substructure of the problem, in such a way that subsolutions are only ever computed once and retrieved as needed for the calculation of the solution. This algorithmic way is known under the name dynamic programming. A simple example for the classical univariate and infinite coin change problem is given in Appendix *B* [App appb]. In the following, we show the adapted pseudocode for the bivariate and finite coin change problem in its crystallographic application, thus taking into account the case distinction for the multipliers of non-fixed and fixed Wyckoff sites (Fig. 2 ▸). The adaptation to the trivariate and finite case including the length *k* as a parameter follows a straightforward procedure (Fig. 3 ▸), as would be the case for any future multivariate generalization, for instance also taking into account chemical degrees of freedom.

In either case, the approach starts with the trivial base case (there is always one possibility for the null tuple), from which it proceeds bottom-up making use of a tabulation implementation of subsolution values cached into either a matrix (bivariate case) or a tensor (trivariate case), which is updated iteratively until the target case is reached and the solution is returned. Equation (29) shows the solution matrix 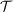 for the case defined by the target tuple 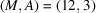 and the multiset of tuples 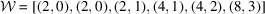 in which the values for 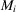 and 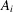 increase along the rows and the columns, respectively. Note that for a more economic display, the matrix is shown in its transposed form, with columns and rows interchanged, such that its upper-left corner represents the matrix element 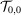 and the lower-right corner represents the matrix element 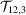 , from which the solution, 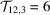 , can be read off: 

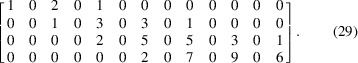

A direct comparison shows that all non-zero entries represent the non-vanishing coefficients as occurring in the polynomial expansion given in equation (24), yet with terms only up to the solution 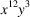 monomial inclusive. Note that the algebraic structure of the Wyckoff positions is reflected in the matrix as well, since the reachable positions are determined by the smallest increments and their parity as observed for the Wyckoff multiplicities and arities. For instance, since all the Wyckoff multiplicities are even numbers, all odd columns of the above matrix are given by the null vector.

A Python implementation of the algorithm for the bivariate case is given in Appendix *C* [App appc], which also contains a *Mathematica* implementation of the generating polynomial approach.

## 3. Complexity of Wyckoff sequences

The aforementioned problem of crystals arose in the context of calculating Shannon entropy based complexity measures for crystal structures, taking into account a crystal structure’s fundamental chemical, combinatorial and coordinational degrees of freedom (Hornfeck, 2020 ▸). Apart from its chemical degrees of freedom – its decoration (colouring) of Wyckoff sites with atoms – a crystal structure is geometrically defined by its combined combinatorial and coordinational degrees of freedom, the collective multiplicities *M* and arities *A* of its occupied Wyckoff positions.

### 3.1. Shannon entropy based complexity measures

In general, a given multiset 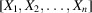 of *n* individual degrees of freedom 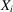 defines a discrete probability distribution 

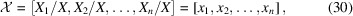

where 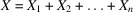 denotes the collective number of degrees of freedom as obtained from the partition of the individual numbers of degrees of freedom. From this a Shannon entropy 

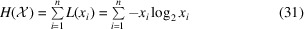

can be obtained. Note that 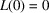 , by definition.

As mentioned before, the general interpretation is that of a system with *X* degrees of freedom on a higher level of structural hierarchy being subdivided into *n* subsystems of 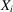 degrees of freedom, each on a lower level of structural hierarchy. For more details of the general theory, the reader is referred to previous work done by the author (Hornfeck, 2020 ▸, 2022*b* ▸).

In particular, a crystal structure’s *M* collective combinatorial degrees of freedom are associated with the multiset 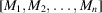 of individual Wyckoff multiplicities 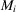 , thereby yielding a fundamental combinatorial Shannon entropy:

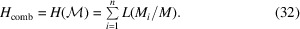

Note that in order to ensure the comparability of combinatorial complexity values between different crystal structures, the values of the Wyckoff multiplicities 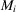 have to refer to a primitive unit cell [although this is really important only for derived complexity values such as the maximal complexity, 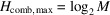 , the normal complexity, 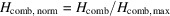 , or the total complexity, 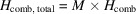 , which have been described by Hornfeck (2020 ▸)].

Now, in the same way, a crystal structure’s *A* collective coordinational degrees of freedom are associated with the multiset 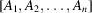 of individual Wyckoff arities 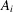 , thereby yielding a fundamental coordinational Shannon entropy 

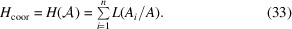

Now, proceeding in a completely analogous manner, a crystal structure’s 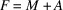 collective configurational degrees of freedom are associated with the combined multiset 

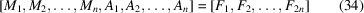

of individual Wyckoff multiplicities 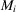 and individual Wyckoff arities 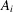 , corresponding to the individual configurational degrees of freedom 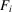 , thereby yielding a composite configurational Shannon entropy 

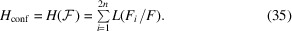

While the association with the combined multiset is natural, it is noteworthy to mention that this does not mean that the corresponding entropies are (simply) additive; on the contrary 

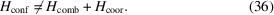

Most importantly, however, by means of the strong additivity property of the Shannon entropy, the configurational entropy is equivalent to the general expression 

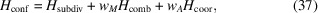

including appropriate weighting factors 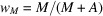 and 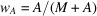 and an additional subdivision (mixing) complexity based on them,

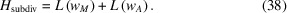

This equivalence now allows an assessment of the relative complexity related to the splitting of a given collective number of degrees of freedom, 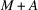 , for the composite system, into individual numbers of degrees of freedom, *M* and *A*, for the fundamental (sub)systems.

**Figure 4:**
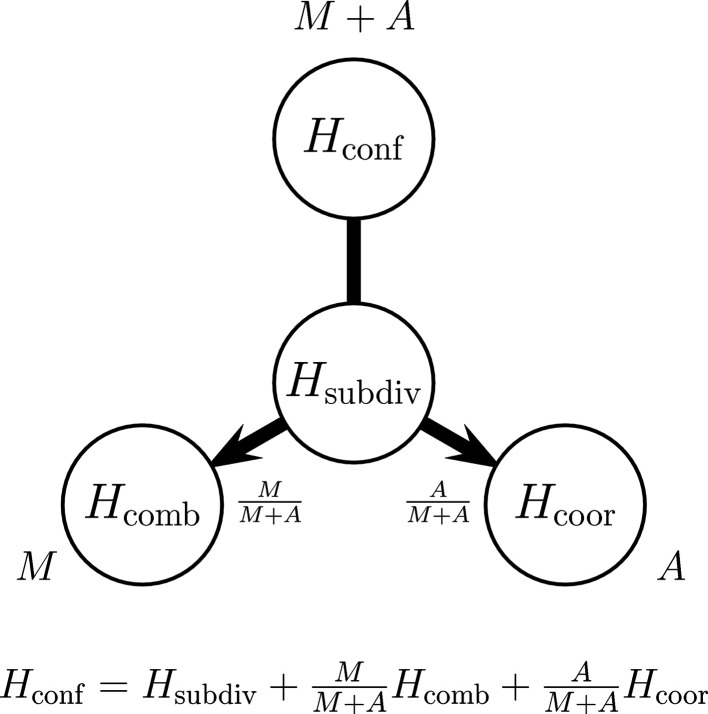
Schematic representation of the relation between the configurational complexity 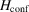 , calculated for 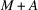 degrees of freedom, and the combinatorial and coordinational complexities, 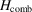 and 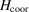 , calculated for *M* and *A* degrees of freedom, respectively. Shown also are the weighting factors 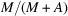 and 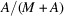 as well as the unit weight contribution of the invariant subdivision complexity 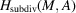 which taken all together sum to the configurational complexity 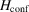 . For a given choice of *M* and *A* the subdivision complexity 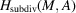 is a constant, while the combinatorial and coordinational complexities, 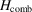 and 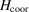 , depend on the respective partitions of *M* and *A* possible for a given space-group type.

The subdivision complexity 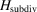 is a Shannon entropy of the weighting factors occurring in equation (37) and for all the combinatorially enumerated cases it is an invariant characterizing the subdivision step 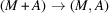 on the higher crystal structure level of hierarchy. It can be seen as connecting the collective treatment of degrees of freedom, within the combined configurational complexity 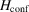 , with the individual treatment of degrees of freedom, within the separated combinatorial and coordinational complexities, 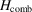 and 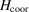 , respectively. This allows for a complexity partition analysis (Hornfeck, 2022*b* ▸). This central importance of the subdivision complexity 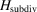 and its relationship to the other complexity measures is graphically depicted in Fig. 4 ▸.

In the same manner, other subdivision complexities exist on the lower Wyckoff position level of hierarchy, characterizing the subdivision steps 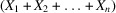 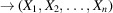 , with *X* denoting either *M* or *A*.

Furthermore, it should be noted that the strong additivity property also holds for the case of maximal Shannon entropies 

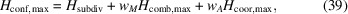

where 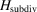 takes on the same value as in equation (37). The maximal entropies are reached in the case of maximal sub­division and thus perfect equidistribution of degrees of freedom (corresponding to maximally expanded partitions 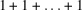 of variable length equal to the number of degrees of freedom). This makes their calculation particularly simple, resulting eventually in 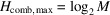 , 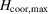 = 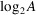 and 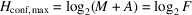 .

Each maximal entropy can also be used in turn to define its corresponding non-maximal entropy, for instance 

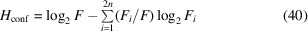

by means of taking the difference between the maximal entropies for the collective (here, *F*) and individual (here, 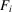 ) degrees of freedom, the latter ones attributed with their appropriate weighting factors 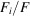 .

**Figure 5:**
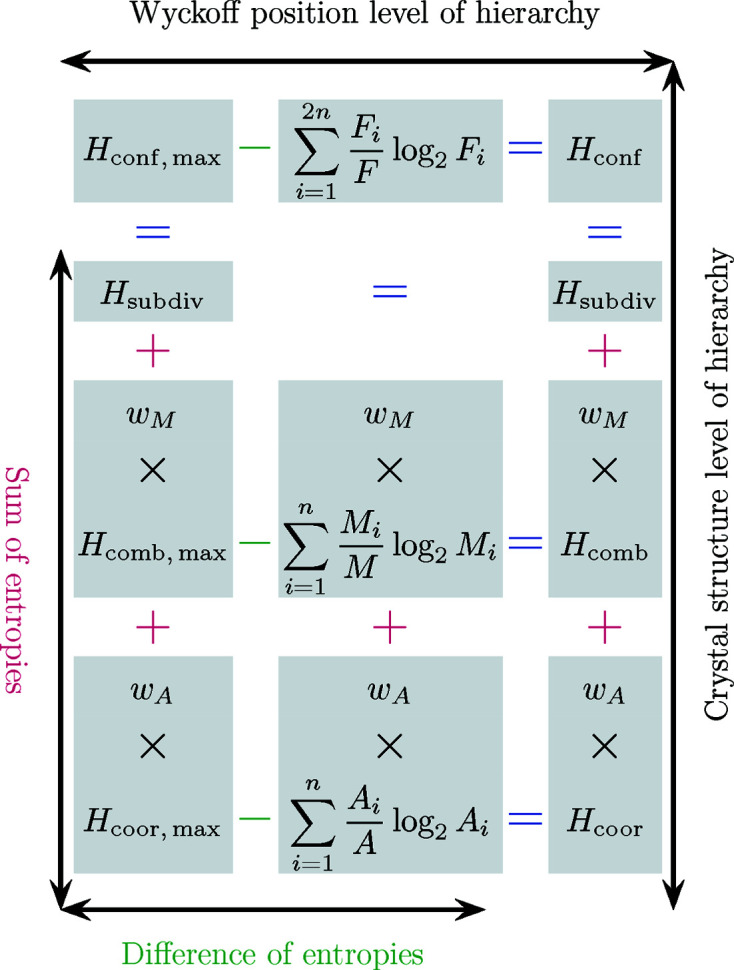
Schematic representation of the interrelation between various maximal and non-maximal entropies (combinatorial, coordinational, configurational) and the subdivision entropy on different levels of structural hierarchy. On the Wyckoff position level of hierarchy the difference of (maximal) entropies defines the conventional non-maximal entropies (from left to right), while on the crystal structure level of hierarchy the (partially weighted) sum of entropies defines the maximal and non-maximal configurational entropy (from bottom to top). Note that the distinction between differences and sums of entropies depends on a deliberate choice of how to distribute terms on either side of the equals sign. In the scheme as presented here, the case for differences of entropies is based on the choice of collecting all maximal entropies together, while distributing collective and individual contributions on opposite sides of the equation could highlight the fact that the non-maximal entropies fulfil the same role as a subdivision complexity on the Wyckoff position level as the subdivision complexity 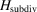 does on the crystal structure level, thus highlighting the applicability of the strong additivity property on both levels of hierarchy (in this case the common weighting factors 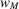 or 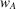 can be omitted on the Wyckoff position level of hierarchy).

All the aforementioned interrelations are depicted in Fig. 5 ▸.

## 4. Combining the combinatorics and complexity of Wyckoff sequences

Some elementary statistical results shall be stated about the magnitude of the collective degrees of freedom to expect, in extreme and on average, and with respect to actual crystal structure data as retrieved from the 20 040 unique Wyckoff sequences compiled in the Pearson’s Crystal Data Crystal Structure Database for Inorganic Compounds (Villars & Cenzual, 2020 ▸).

The minimal observed combinatorial and coordinational degrees of freedom are, as determined for the individual, independent distributions, 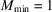 , 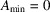 , the maximal are 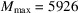 , 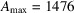 , the mean values are 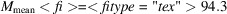 , 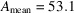 , the median values are 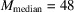 , 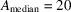 , and the mode values are 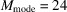 , 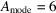 , respectively.

Our combinatorial result now tells us how many of these partitions, being solutions to a combinatorial problem restricted by crystallographic symmetry, can be realized for a given space group, given the fixed number of degrees of freedom 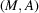 and, potentially, the length *k* of the Wyckoff sequence. In the following we will illustrate this by performing explicit calculations for three examples.

It should be noted that the examples given here were chosen such that the discussion of the results could be made explicit, with the full set of potential Wyckoff sequence solutions being listed, and with some actual representatives being found among the known crystal structures. In general, the number of potential solutions might exhibit a rapid growth (combinatorial explosion), while the number of actual representatives lags behind greatly.

### 4.1. Example 1: space-group type *P* 42_1_ *m* (No. 113) revisited

As our first example, we revisit the case of space-group type 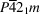 (No. 113). Table 1 ▸ compiles some values of specific Shannon entropies for the Wyckoff sequences given in this example, taking into account the combinatorial, coordinational and configurational degrees of freedom, highlighting the constant term 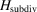 , with the other Shannon entropies related according to their strong additive sum:

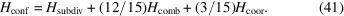




A search in the Pearson’s Crystal Data Crystal Structure Database for Inorganic Compounds (Villars & Cenzual, 2020 ▸) for the space-group type 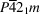 (No. 113) reveals a total of 759 Wyckoff sequences for which crystal structure prototypes have been assigned. Reducing for multiple entries arising from multiple crystal structure determinations sharing the same prototype yields 79 entries with a unique Wyckoff sequence/prototype combination. (Notably, 538 entries share the same Wyckoff sequence 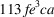 , with seven distinct prototypes associated to it, of which the *tP*24–Ca_2_MgSi_2_O_7_ prototype alone occurs 307 times, thereby explaining the heavy degree of reduction observed.) Reducing again for distinct prototypes sharing the same Wyckoff sequence yields 63 unique Wyckoff sequences.

Of these, 55 correspond to a unique pair 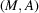 of combinatorial and coordinational degrees of freedom, with six pairs occurring two times, and one pair, 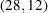 , occurring three times. The associated Wyckoff sequences and prototypes are: 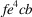 (Th_2_Se_5_ prototype), 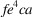 [(Ca_0.25_La_0.75_)_2_Ga_3_O_7.25_ prototype] and 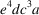 (Ce_2_CoAl_7_Ge_4_/LiSmTiO_4_ prototypes).

On the basis of this work, one obtains a larger number of 44 possible Wyckoff letter sequence solutions [based on the Wyckoff multiplicities and arities, compare equation (21)], which can be constructed from an exhaustive exploration of a search space of much larger size of 11 648 multiplier choices (representatives with prototypes marked by an asterisk): 

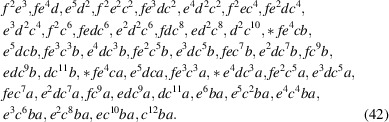

While the combinatorial approach is non-constructive, it might often suffice to only know the number of possible solutions.

### 4.2. Example 2: space-group type *Fm* 3 *m* (No. 225)

As another example, we consider the cubic space-group type 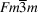 (No. 225) encompassing a total of 12 distinct Wyckoff positions of the following multiplicities and arities: 

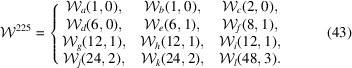

Note that the multiplicities stated here are those for a reduced (primitive) unit cell, while the multiplicities for an *F*-centred unit cell would be four times larger. Again, all of the four possible values for the Wyckoff arity are present in this example, in addition to seven distinct values for the Wyckoff multiplicity. This example was chosen because it partly corresponds to actual crystal structures.

Now, for the given choice of 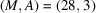 degrees of freedom one finds 17 matching Wyckoff letter sequences: 

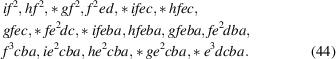

Here, the seven sequences preceded by an asterisk are realized as crystal structures. A compilation of their complexity values, which are interrelated according to 

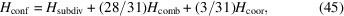

is given in Table 2 ▸. Here, only the combinatorial contribution 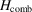 is responsible for the variable amount of configurational complexity 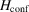 , the other terms, in particular the value for 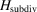 , being constant.

If one performs the same calculation with the additional restriction of the Wyckoff sequence length to the value 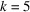 one obtains the number of four solutions, which the reader can easily check on the list given above.

### 4.3. Example 3: space-group type *Cmcm* (No. 63)

As a final example, we consider the orthorhombic space-group type *Cmcm* (No. 63) encompassing a total of eight distinct Wyckoff positions of the following multiplicities and arities: 

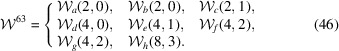

Note that the multiplicities stated here are those for a reduced (primitive) unit cell, while the multiplicities for a *C*-centred unit cell would be two times larger. Again, all of the four possible values for the Wyckoff arity are present in this example, in addition to three distinct values for the Wyckoff multiplicity.

Now, for the given choice of 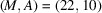 degrees of freedom, one finds 67 matching Wyckoff letter sequences: 

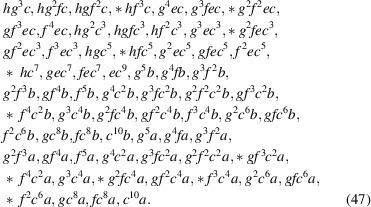

Here, the 11 sequences preceded by an asterisk are realized as crystal structures. A compilation of their complexity values, which are interrelated according to 

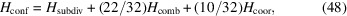

is given in Table 3 ▸. Here, both the combinatorial and coordinational degrees of freedom are responsible for the variable amount of configurational complexity 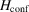 .

## 5. Conclusion

As a contribution to the combinatorics of Wyckoff sequences, we have presented two methods to calculate their number for a fixed space group, given a pair of combinatorial and coordinational total degrees of freedom, and, optionally, their length. The first method is based on a generating polynomial approach (see Sections 2.4 and 2.7 for the key results), while the second makes use of a dynamic programming algorithm (Section 2.8). While the generating polynomial approach appears to be conceptually easier to understand, the dynamic programming algorithm is considerably better in its computational performance. The methods have been exemplified on cases of ideal and actual crystal structures with invariant subdivision complexity and variable configurational complexity in the sense of Hornfeck (2020 ▸), thus relating the combinatorics of Wyckoff sequences to the complexities of crystal structures.

## Figures and Tables

**Figure 6:**
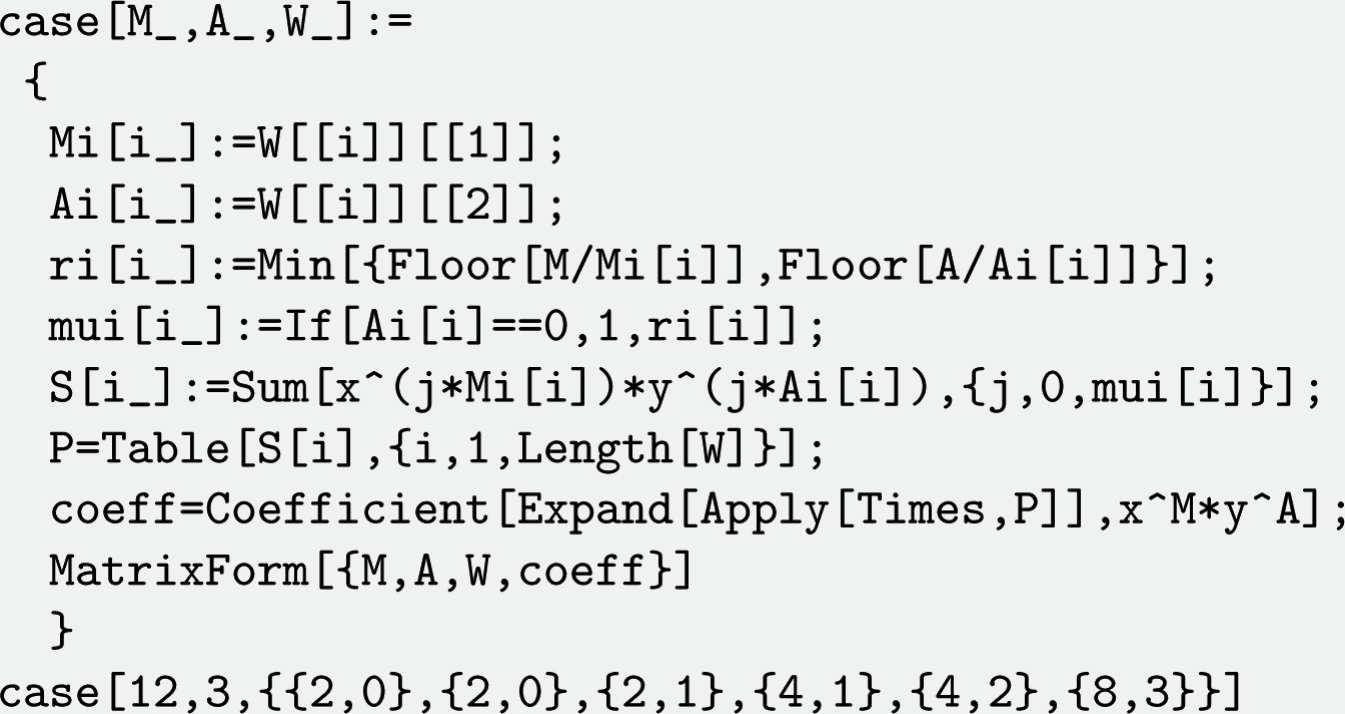
Generating polynomial approach (*Mathematica*).

**Figure 7:**
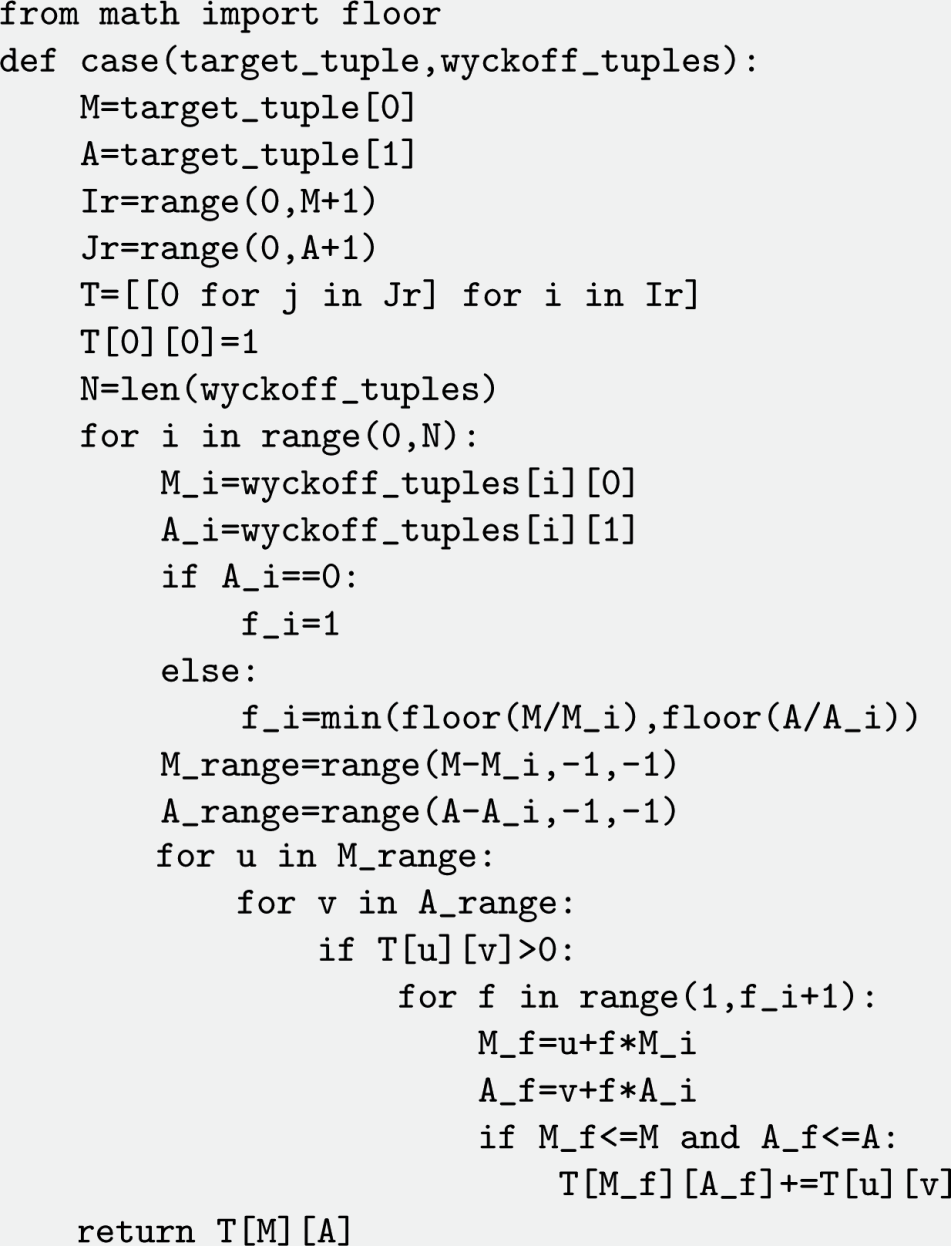
Dynamic programming approach (Python). Here, the target tuple is given as 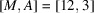 and the Wyckoff tuples are given as 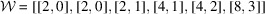 .

**Table 1:** Configurational complexities for the six possible Wyckoff sequences (of length *k*) with 12 combinatorial and three coordinational degrees of freedom of space group type number 113 Shannon entropies are stated in units of bit per freedom.

Wyckoff sequence	*k*	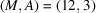	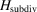	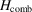	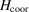	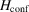
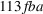	3	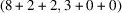	0.722	1.252	0.000	1.723
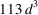	3	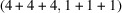	0.722	1.585	1.585	2.307
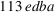	4	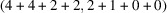	0.722	1.918	0.918	2.440
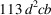	4	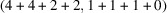	0.722	1.918	1.585	2.574
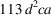	4	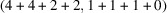	0.722	1.918	1.585	2.574
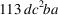	5	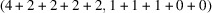	0.722	2.252	1.585	2.840

**Table 2:** Configurational complexities for the seven possible Wyckoff sequences (of length *k*) with 28 combinatorial and three coordinational degrees of freedom of space group type number 225 which have realizations as crystal structures in nature Shannon entropies are stated in units of bit per freedom. Information regarding the structure type for which the Wyckoff sequence is realized is taken from Pearson’s Crystal Data Crystal Structure Database for Inorganic Compounds (Villars & Cenzual, 2020 ▸). If more than one structure type is associated with the same Wyckoff sequence a selection has been made.

Wyckoff sequence	*k*	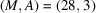	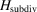	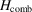	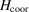	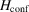	Structure type
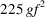	3	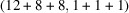	0.459	1.557	1.585	2.018	Y_0.25_Bi_0.75_O_1.5_
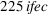	4	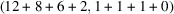	0.459	1.788	1.585	2.227	KY_3_F_10_, Zr_3_PbO_4_F_6_
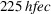	4	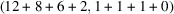	0.459	1.788	1.585	2.227	Y_0.27_Bi_0.73_O_1.5_
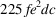	5	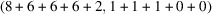	0.459	2.217	1.585	2.615	Cs_3_Re_3_S_4_I_4_
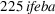	5	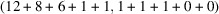	0.459	1.860	1.585	2.292	(Ag_0.5_Pd_0.5_)_11_Se_3_
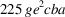	6	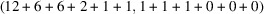	0.459	2.092	1.585	2.501	Cr_0.8_Mn_0.2_Mn(CN)_6_(H_2_O)_4_
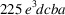	7	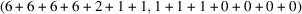	0.459	2.520	1.585	2.888	K_0.04_(VO)Co_0.88_(CN)_3.8_(H_2_O)_1.1_

**Table 3:** Configurational complexities for the 11 possible Wyckoff sequences (of length *k*) with 22 combinatorial and ten coordinational degrees of freedom of space group type number 63 which have realizations as crystal structures in nature Shannon entropies are stated in units of bit per freedom. Information regarding the structure type for which the Wyckoff sequence is realized is taken from Pearson’s Crystal Data Crystal Structure Database for Inorganic Compounds (Villars & Cenzual, 2020 ▸). If more than one structure type is associated with the same Wyckoff sequence a selection has been made.

Wyckoff sequence	*k*	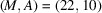	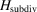	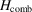	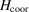	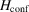	Structure type
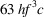	5	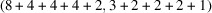	0.896	2.187	2.246	3.101	SrGe_5.5_
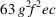	6	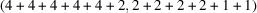	0.896	2.550	2.522	3.438	K_2_Zn_5_As_4_
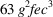	7	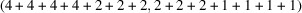	0.896	2.732	2.722	3.625	[UO_2_]Cl_2_[NH_3_]_6_
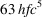	7	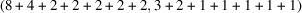	0.896	2.550	2.646	3.476	Yb_2_Mn_0.33_Si_3.67_
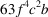	7	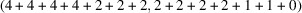	0.896	2.732	2.522	3.562	K_2_Cu_3_US_5_
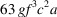	7	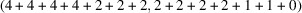	0.896	2.732	2.522	3.562	Zn(Zn_0.45_Co_0.55_)Co_3_Sn_4_
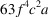	7	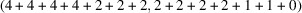	0.896	2.732	2.522	3.562	CaFe_4_O_6_
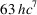	8	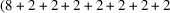 , 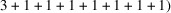	0.896	2.732	2.846	3.664	KNaOs[NO][F_5_][H_2_O]
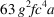	8	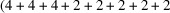 , 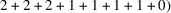	0.896	2.914	2.722	3.750	β-U_3_O_8_
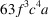	8	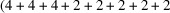 , 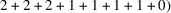	0.896	2.914	2.722	3.750	Cs_2_Cu_5_Se_4_
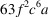	9	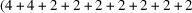 , 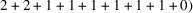	0.896	3.096	2.922	3.938	Sr_2_Ta_2_O_7_

## Appendix A Number of Wyckoff sequences of length *k*

The number of Wyckoff sequences of length *k* is given as

in which ν and φ, respectively, denote the number of non-fixed and fixed Wyckoff positions of a given space group [equation (7) in Hornfeck (2022*a* ▸)]. In the worst case  and  , thus

gives the number of distinct Wyckoff sequences up to  inclusive.

## Appendix B The classical coin change problem

Assume you are in the possession of the following multiset of coins: three coins of value  , four coins of value  , two coins of value  . How many ways exist to pay a total price of value  ?

Let  denote the individual sum of coins of value *v*, hence

The values which each individual sum may take are determined and limited in number by the number of available coins of each nominal value. Thus, one gets

as all possible values. Now, the trick is that one identifies these values with the exponents of a couple of generating polynomials, one for each type of coin, namely

in which  by definition. From these individual polynomials one forms their product

and analyses its expansion

The coefficient of  in  gives the desired solution, here it is equal to four, describing the four cases (i)  , (ii)  , (iii)  and (iv)  , all amounting to 12.

A generalization is possible, in case the number of coins available for all given values *v* is assumed to be infinite. Then, the generating polynomials are replaced by the respective generating functions of a geometric series,

and the solution to the problem is given by their product

in the same way as before, namely as the value of the coefficient  in  , in which *V* denotes the total price. In the infinite case the result is  , necessarily containing at least as many or even more solutions than were found in the finite case. In this general form the problem has been addressed by Pólya (1956 ▸). A closed form solution for the univariate infinite case has been described by Graham *et al.* (1994 ▸, pp. 327–330 and pp. 344–346).

Note that

is the generating function of the number-theoretic integer partition function (Alfonsín, 2005 ▸, p. 71), and our problem asks for the number of restricted partitions into specified, possibly repeated parts (a finite or infinite number of coins with a finite set of distinct denominations). In this context the number of non-negative integer solutions is known as Sylvester’s denumerant (Sylvester, 1857 ▸) which has a rich history in number theory, partly due to the difficulty of obtaining exact results (Alfonsín, 2005 ▸, ch. 4).

Finally, it should be emphasized that an elegant method of solving this problem in practice is given by the concept of dynamical programming. Since the problem again and again reduces to simpler subproblems of the same kind, the solutions of these subproblems can be stored whenever they occur for the first time. Moreover, using a bottom-up approach (top-down would be equally feasible), the solutions for sub­problems can be systematically generated from the already known solutions. For this purpose the function[Chem scheme1]

here given in a Python implementation is initialized with a result vector of length  of the form  . This result vector is subsequently updated in a separate loop for every coin value. The final value at position *n* of the result vector then is the solution to the (infinite) coin problem described before (for  the result is equal to 13).

## Appendix C Code examples

Figs. 6 ▸ and 7 ▸ give working examples for the calculation of the number of Wyckoff sequences with the same subdivision complexity by means of the generating polynomial and dynamic programming approaches. The input values are taken from the example described in Section 2.6, with the output being equal to six.
